# Pharmacological Mechanisms of Kirenol against Ovarian Carcinoma: A Network Pharmacology and Experimental Validation Study *In Vitro*

**DOI:** 10.2174/0113862073289977240216075724

**Published:** 2024-02-26

**Authors:** Xiaoling Mao, Hong Zhu, Jun Gao, Shixin Lin, Yin Bao, Mingyue Zhang, Huan Yang

**Affiliations:** 1 Jiangxi University of Chinese Medicine, Nanchang, Jiangxi 330006, P.R. China;; 2 Department of Gynecologic Oncology, Jiangxi Cancer Hospital, Nanchang, Jiangxi 330029, P.R. China;; 3 Department of Jiangxi Key Laboratory of Translational Research for Cancer, Jiangxi Cancer Hospital, Nanchang, Jiangxi 330029, P.R. China;; 4 Department of Obstetrics and Gynecology, West China Second University Hospital, Sichuan University, Chengdu 610041, P.R. China;; 5 Department of Third Clinical Medical College of Nanchang University, Third Affiliated Hospital of Nanchang University, Nanchang, Jiangxi 330008, P.R. China

**Keywords:** Kiren, ovarian carcinoma, PI3K/AKT/CDK4, cell cycle, apoptosis, metastasis

## Abstract

**Background:**

Ovarian carcinoma is an aggressive gynecological malignancy. Kirenol, a diterpene compound, has recently gained attention for its potential anticancer properties. However, its exact anti-tumor mechanism remains largely unexplored.

**Objective:**

In this study, we explored the inhibitory effects of Kirenol on ovarian cancer using network pharmacology and *in vitro* experiments and elucidated its underlying mechanisms.

**Methods:**

Through the utilization of molecular docking, we established a network of protein-protein interactions (PPI), which unveiled CDK4 as an essential target. Additionally, gene enrichment and pathway analysis highlighted the significance of the PI3K/AKT pathway. The viability of ovarian cancer cells and normal ovarian epithelial cells was evaluated using CCK8 assays to determine the effect of Kirenol. Following *in vitro* tests, cell colony formation, wound healing, flow cytometry, and Western blotting were conducted to assess its impact on cell proliferation, metastasis, apoptosis, and the cell cycle.

**Results:**

Kirenol significantly reduced the viability of ovarian cancer cells (SKOV3 and A2780) compared to normal ovarian epithelial cells (IOSE-80). Moreover, Kirenol efficiently suppressed the growth and movement, caused a cell cycle halt, and stimulated programmed cell death in SKOV3 and A2780 cells. Through molecular analysis, it was observed that Kirenol increased the expression of Bax while decreasing the expression of MMP2, MMP9, and Bcl-2. It also attenuated the phosphorylation of PI3K, AKT, and RB and downregulated CDK4 and CCND1 expression. Notably, co-treatment with the PI3K pathway inhibitor LY294002 enhanced the inhibitory effect of Kirenol on ovarian cancer cells.

**Conclusion:**

In summary, the combined results of our network pharmacology analysis and *in vitro* tests emphasized that Kirenol hinders the growth of ovarian cancer cells, causes cell cycle arrest, enhances apoptosis, and hampers migration, possibly by regulating the PI3K/AKT/CDK4 signaling pathway.

## 1 INTRODUCTION

Ovarian carcinoma (OC) is an extremely deadly gynecological cancer, placing it in the top 10 female malignancies in terms of both occurrence and fatality. Based on data provided by the International Agency for Research on Cancer, OC had around 313,959 newly detected instances in 2020. The number of OC-related deaths increased significantly to 207,252, with China alone accounting for 55,000 new cases and 37,000 fatalities [1]. Presently, the main focus of OC treatment involves surgical procedures, chemotherapy, and radiotherapy. However, these treatment methods often encounter challenges due to adverse effects and multidrug resistance. Despite the considerable progress made in the use of PARP inhibitors for OC treatment, the disease continues to be extremely aggressive, resulting in a devastating prognosis where approximately 60% of stage III patients and 80% of stage IV patients die from the disease within a period of 5 years [2]. In light of these challenges, the imperative to develop novel and more effective drugs to combat OC becomes evident. *Siegesbeckia orientalis*, an Asteraceae herb native to Central China, has been traditionally employed in treating arthritis and hypertension [3].

Of particular interest is Kirenol, an ent-pimarane type diterpenoid derived from the foliage of *Siegesbeckia orientalis*, with the molecular formula C20H34O4. Kirenol displays a wide array of pharmacological characteristics, encompassing anti-inflammatory, antioxidant, and anticancer effects. Notably, Kirenol has shown promising results as a therapeutic agent for inflammatory disorders, acting on the AMPK-mTOR-ULK1 pathway to attenuate lung inflammation [4]. Furthermore, it efficiently reduces inflammation in fibroblast-like synoviocytes of rheumatoid arthritis both in laboratory settings and in living organisms [5]. Kirenol has the ability to alleviate Doxorubicin-induced cardiac hypertrophy by blocking the Nrf2 signaling through the PI3K/AKT pathway [6]. It also hinders osteoclastogenesis by inhibiting RANKL and offers protection against ovariectomy-induced osteoporosis by suppressing the Ca2+-NFATc1 and Cav-1 signaling pathways [7]. More recently, Kirenol has shown promise in alleviating melanogenesis induced by MITF by inhibiting intracellular ROS production [8]. Moreover, the anti-tumor capabilities of Kirenol also encompass the modulation of the PI3K/AKT and MAP kinase pathways, leading to the suppression of human thyroid cancer cell proliferation [9].

Consequently, this study aimed to investigate the anti-cancer effectiveness of Kirenol on ovarian cancer cells and uncover the molecular mechanisms involved. Our findings offer encouraging prospects for the treatment of ovarian cancer in the future.

## 2 MATERIALS AND METHODS

### 2.1 Network Pharmacology

#### 2.1.1 Prediction and Screening of Kirenol Targets

The SwissTarget Prediction database (http://www.swisstargetprediction.ch/) was utilized to predict the potential target of Kirenol. This online platform is renowned for its capability to predict small-molecule drug targets. The screening criteria employed were based on the following parameters: “high” gastrointestinal absorption (GI absorption) and positive results in at least 2 out of the following criteria: Lipinski, Ghose, Veber, Egan, and Muegge rules. These stringent criteria ensure a robust and reliable prediction of Kirenol's target, enhancing our understanding of its pharmacological mechanism of action.

#### 2.1.2 Prediction of OC Targets

Several well-known databases, such as GeneCards, Drugbank, PharmGKB, TTD, and OMIM, were searched using the terms 'Ovarian Cancer,' 'Ovarian Neoplasms,' 'Ovarian Carcinoma,' and 'Ovarian Tumor' to identify important targets for ovarian cancer treatment. Subsequently, the retrieved targets from these databases were amalgamated, and duplicate entries were meticulously eliminated to ensure a comprehensive and non-redundant collection of core targets for further analysis in the context of ovarian cancer therapy.

#### 2.1.3 Protein-protein Interaction Network

The intersection of drug targets and disease targets presents a valuable approach to identifying potential targets for Kirenol in the context of ovarian cancer. In order to build the PPI network, the anticipated targets were entered into the String database (https//string-db.org/), with the species specified as 'Homo sapiens', and the following criteria applied: 'high confidence > 0.9' and 'hide disconnected nodes in the network'. Default settings were maintained for other parameters during network construction. Afterward, the PPI network's topological parameters were examined using the CytoNCA plug-in in Cytoscape 3.9.0 software. These parameters included betweenness centrality (BC), closeness centrality (CC), degree centrality (DC), eigenvector centrality (EC), network centrality (NC), and local edge connectivity (LAC). Additionally, the topological parameters of the PPI network were evaluated using a screening criterion, where values surpassing the median value of all nodes were deemed significant. Targets meeting this criterion were subjected to two consecutive screenings, resulting in the identification of core targets of Kirenol in ovarian cancer. These core targets hold potential significance for further exploration and evaluation as therapeutic candidates in the treatment of ovarian cancer.

#### 2.1.4 Molecular Docking

The PubChem database provided the 2D structures of the key compounds. The predictive structures of the main targets, including MTOR (PDB ID 1aue), EGFR (PDB ID 1ivo), IL6 (PDB ID 1alu), JAK2 (PDB ID 2b7a), MAP2K1 (PDB ID 1s9j), MMP9 (PDB ID 1gkc), CDK4 (PDB ID 2w96), and AR (PDB ID 1e3g), were obtained from the Protein Data Bank (www.rcsb.org/). These protein structures were then prepared for molecular docking using PyMol 2.3.4 (https//pymol.org/). To prepare them, water molecules were removed, nonpolar hydrogen atoms were added to the structures, and the processed files were saved in PDBQT format. To assess the strength of the binding interactions, the binding energy, which quantitatively measures the affinity of the molecular compound to its target, was utilized. Molecular docking was performed using Autodock Vina 1.1.2 (https//vina.scripps.edu/), a computational technique that enables the docking of ligands to their corresponding target molecules. The method provided a valuable understanding of the potential connections between molecules, illuminating the potential molecular mechanisms that explain the observed effects of the core compounds on the chosen targets.

#### 2.1.5 GO Enrichment Analysis and KEGG Pathway Enrichment Analysis

The predicted targets were subjected to comprehensive analysis using the biological information annotation database DAVID (http://david.nifcrf.gov/). To determine the functions of the identified targets, enrichment studies were carried out, aiming to clarify the Gene Ontology (GO) function and the Kyoto Encyclopedia of Genes and Genomes (KEGG) pathway linked to them. By employing this method, our objective was to acquire an understanding of the biological mechanisms and signaling routes in which the anticipated targets are involved, illuminating the potential ways in which Kirenol operates in the context of treating ovarian cancer.

## 3 EXPERIMENTAL SECTION

### 3.1 Cell Culture

For experimental purposes, iCell Bioscience Inc. (located in Shanghai, China) supplied the IOSE-80 cell line, which is a human normal ovarian epithelial cell line, as well as the SKOV3 and A2780 cell lines, which are human ovarian cancer cell lines. RPMI1640, which was supplemented with 10% fetal bovine serum (BI) (Kmaels (Shanghai) Biotechnology Co., Ltd., Shanghai, China) and 1% penicillin/streptomycin (P/S), was used as the culture media for A2780 and IOSE-80 cells. The cells were incubated at 37°C in a humidified atmosphere containing 5% CO_2_. The SKOV3 cells were cultured in McCoy's 5A medium (Boster Biological Technology Co., Ltd., Wuhan, China), while the remaining culture conditions were unchanged for the mentioned cells. Kirenol (purity ≥99.6%) and LY294002 (purity ≥ 99.76%) were obtained from Tsbiochem (#T4S1943; #T2008). Furthermore, 500 mmol/L of dimethyl sulfoxide (DMSO) was used to dissolve these compounds. Various concentrations of Kirenol were applied to the cells in a fresh medium, while the control group was treated with DMSO at an equivalent volume to the high-dose group for comparison.

### 3.2 Cell viability Assay

In 96-well plates, SKOV3, A2780, and IOSE-80 cells were seeded individually at densities of 2000, 1000, and 1000 cells per well, correspondingly. Following a 6-hour incubation period, the cells were exposed to different levels of Kirenol-infused solution (0, 100, 200, 300, 400, 500, 600, and 700 μmol/L) and subsequently cultured for 72 hours. To assess cell viability, the CCK-8 assay kit (Abbkine Scientific Co., Ltd., Wuhan, China) was utilized, with a mixture of complete medium and CCK-8 at a ratio of 9:1. Afterward, the cells were placed in an incubator set at a temperature of 37°C for a duration of 60 minutes. Afterwards, the microplate reader was used to measure the optical density (OD) at 450 nm in order to calculate the IC50 value. Furthermore, the cells were placed onto a 96-well plate and given 6 hours to adhere. Subsequently, varying concentrations of kierenol were introduced to the initial medium (0, 100, 200, 300, 400, 500, 600, and 700 μmol/L). In the original medium, a mixture of CCK-8 was introduced at 24, 48, and 72 hours. Following an additional incubation of the 96-well plate at 37°C for 1 hour, the optical density was assessed at 450 nm. These trials aimed to assess the toxic impact of Kirenol on SKOV3, A2780, and IOSE-80 cells, as well as to ascertain the compound's IC50 in these cell lines. The CCK-8 assay allowed for the assessment of cell viability at different time points and Kirenol concentrations, providing valuable insights into its potential anti-cancer activity.

### 3.3 Colony Formation Experiment

Before conducting the clonogenic assay, SKOV3 and A2780 cells underwent a 72-hour pre-treatment with different doses of Kirenol, namely 0, 100, 150, and 200 μmol/L for SKOV3 cells, and 0, 100, 200, and 300 μmol/L for A2780 cells. After undergoing this pretreatment, 500 cells were placed per Petri dish and allowed to proliferate for a period of ten to fourteen days. Following the incubation phase, the cells were treated with a 0.2% crystal violet solution for one hour and then fixed in 100% methanol for 30 minutes. Afterward, the cells were extensively rinsed and allowed to air dry. To evaluate the extended proliferative capacity and viability of SKOV3 and A2780 cells following exposure to varying concentrations of Kirenol, the clonogenic assay was conducted. By utilizing this experimental method, it became possible to observe the formation of colonies and gather significant data regarding the compound's capacity to hinder cell growth and colony formation. Consequently, this aids in assessing its potential as an anti-cancer agent.

### 3.4 Wound-healing Assay

In 6-well plates, SKOV3 and A2780 cells were cultivated to form a monolayer. Subsequently, the cell monolayers were intentionally scratched using a 200 μl pipette tip to create wounds. Following that, the cells underwent a thorough PBS wash. Subsequently, SKOV3 and A2780 cells were subjected to varying doses of Kirenol, specifically 0, 100, 150, and 200 μmol/L for SKOV3 cells and 0, 100, 200, and 300 μmol/L for A2780 cells. Microscopy was used to closely monitor and record the wound healing process at 0 hours (right after scratching) and at 48 hours, with the capture of photographs. The migratory capability of SKOV3 and A2780 cells in response to Kirenol treatment was assessed through the wound healing assay. By observing and documenting the closure of the scratched wounds over time, this assay provides valuable insights into the compound's potential to impede cell migration and motility, which are important aspects of cancer cell metastasis and invasion.

### 3.5 Cell Cycle by Flow Cytometry

The cells SKOV3 and A2780 underwent a pretreatment for 72 hours using different amounts of Kirenol, namely 0, 100, 150, and 200 μmol/L for SKOV3 cells and 0, 100, 200, and 300 μmol/L for A2780 cells. Cells were separated, centrifuged, and then resuspended in PBS that had been chilled beforehand following the pretreatment phase. Afterward, they were placed overnight at a temperature of -4°C in the presence of 70% ethanol. To conduct cell cycle analysis, the cells that had been fixed were treated with PI/RNase staining buffer (Keygentec, Jiangsu, China). Each flow cytometer tube received 500 μL of cell suspensions and was then allowed to stain in the dark for a duration of 30 minutes. BD Biosciences equipment was utilized in flow cytometry to determine the distribution of cells in various stages of the cell cycle (G0/G1, S, and G2/M) using flow cytometry. The examination provides significant insights into the impact of Kirenol on the advancement and multiplication of SKOV3 and A2780 cells, shedding light on its potential as a controller of cellular growth and a potential treatment for ovarian cancer.

### 3.6 Apoptosis by Flow Cytometry

Apoptosis was evaluated using flow cytometry analysis with the Annexin V-FITC/PI Apoptosis Detection Kit (Keygentec, Jiangsu, China). Pretreatment of SKOV3 and A2780 cells involved exposing them to varying concentrations of Kirenol for 72 hours. The concentrations used were 0, 100, 150, and 200 μmol/L for SKOV3 cells and 0, 100, 200, and 300 μmol/L for A2780 cells. Following the initial treatment stage, the cells were isolated using trypsin without EDTA, spun at a speed of 800 revolutions per minute for a duration of five minutes, and subsequently reconstituted in pre-chilled PBS. After discarding the supernatant, the cells were suspended in 300 μL of binding buffer, along with 3 μL of Annexin V-FITC and 3 μL of Propidium Iodide reagent. Subsequently, the cell suspension was incubated in darkness for a duration of 15 minutes. Flow cytometry, utilizing BD Biosciences equipment, was employed to measure and analyze apoptosis. This experimental setup enabled the detection and quantification of apoptotic events in SKOV3 and A2780 cells upon exposure to different concentrations of Kirenol. The use of Annexin V-FITC and Propidium Iodide allowed for the distinction between apoptotic cells and both live and dead cells. This distinction provides valuable information regarding the compound's ability to induce apoptosis and its potential as a therapeutic agent for ovarian cancer.

### 3.7 Western Blot

Pretreatment of SKOV3 and A2780 cells involved exposing them to varying concentrations of Kirenol for 72 hours. The concentrations used were 0, 100, 150, and 200 μmol/L for SKOV3 cells and 0, 100, 200, and 300 μmol/L for A2780 cells. In the following response experiment, the cells were treated for a further 48 hours with either LY294002 (10 μmol/L) alone or in combination with Kirenol (100 μmol/L). Cell lysates were prepared by adding Phenylmethylsulphonyl fluoride (PMSF; Solarbio, Beijing, China) to RIPA lysis buffer (Beyotime Biotech, Jiangsu, China) in a ratio of 100:1. Following cell lysis, the protein concentration was measured using a BCA Protein assay Kit (Beyotime Biotechnology Co., LTD., Shanghai, China). Protein samples were then denatured by heating at 99°C for 15 minutes. Afterward, a rapid preparation kit for polyacrylamide gel electrophoresis (PAGE) from Servicebio Technology Co., Ltd., located in Wuhan, China, was employed to separate and resolve the protein samples. Then, the protein samples were transferred onto polyvinylidene difluoride (PVDF) membranes provided by Bio-Rad, a company based in Hercules, California, United States. The PVDF filters were treated with 5% skim milk for 2 hours at room temperature, followed by an overnight incubation at 4°C with primary antibodies, and then incubated with secondary antibodies for 2 hours at room temperature. In the end, the protein bands were observed using a Tanon 5200 Chemiluminescence Imaging System, and signal detection was performed with ECL Western blotting substrate (Tianneng Life Science Co., LTD., Shanghai, China). ACTIN served as the internal control for protein normalization. The primary antibodies used were ACTIN (Immunoway, China), Matrix metalloproteinase (MMP)-2 (Abcam, Cambridge, United States), Bcl-2 (Abmart, China), Bax (Abmart, China), PI3K (Abmart), Phospho-PI3K (Tyr467/199) (Abmart), AKT (Abmart), Phospho- AKT (Ser473) (Abmart), CCND1 (Immunoway), CDK4 (Abcam), and Phospho- RB (Ser 807) (Abcam). To investigate the effects of Kirenol on SKOV3 and A2780 cells, a series of experiments were conducted using Image J (version 1.51J8) to measure the intensity of the blot. The experiments also aimed to understand the potential molecular mechanisms underlying the effects of Kirenol by analyzing protein expression and alterations in response to Kirenol treatment, either alone or in combination with LY294002. Through Western blotting analysis, it became possible to examine alterations in proteins and the signaling pathways implicated in the reaction to the therapy.

### 3.8 Statistical Analysis

GraphPad Prism version 8.0 (GraphPad Software, La Jolla, CA, United States) was utilized for conducting statistical analyses. The information is displayed as the average plus the standard deviation (SD). To compare two groups, the t-test was utilized, while groups were compared with each other using one-way ANOVA. Statistical significance was assigned to p-values that were below 0.05. In order to ensure the dependability and consistency of the findings, every trial was conducted on three separate occasions.

## 4 RESULTS

### 4.1 Potential Targets for Kirenol Therapy in OC

**Fig. (1):**
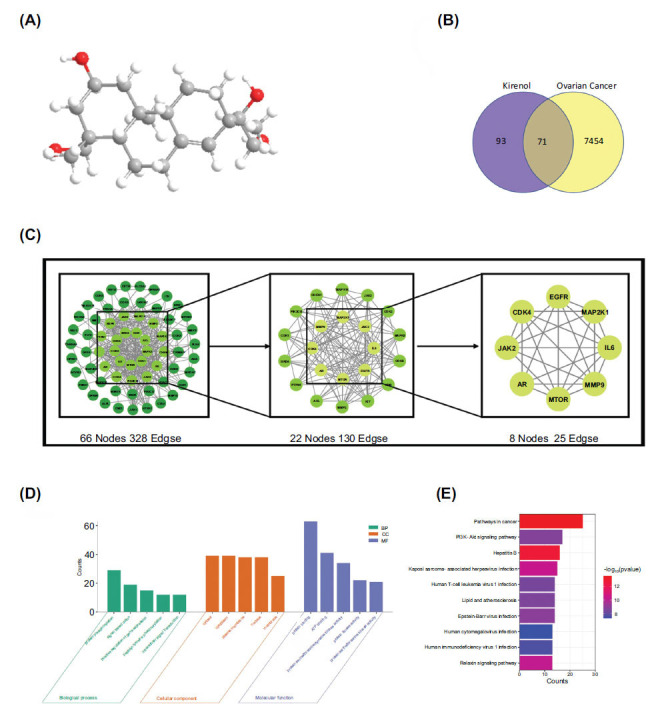
Analysis of Kirenol's enrichment and target prediction in ovarian cancer. (**A**) The Kirenol 3D structure. (**B**) There are 71 overlapping targets between ovarian cancer and Kirenol. (**C**) The PPI network created using the STRING database and had the highest confidence score (0.900). Based on the intersection genes, 66 protein nodes were obtained in this instance (C-1). After screening by CytoNCA plugin for the first time, a total of 22 protein nodes were obtained (C-2), and the first 8 proteins were extracted according to CytoNCA plugin for the second time (C-3). (**D-E**) Seventy-one overlapping targets by DAVID database for GO and KEGG analysis.

The 3D configuration of Kirenol was obtained from the PubChem database (Fig. **1A**). Subsequently, a comprehensive target prediction using the SwissTargetPrediction database identified 93 potential targets for Kirenol. Extensive searches were performed in the Genecards, Drugbank, PharmGKB, TTD, and OMIM databases to clarify its significance in OC. This led to the discovery of 7454 targets linked to OC. By examining the intersection of drug targets and disease targets, a more precise compilation of 71 specific Kirenol targets for OC was obtained (Fig. **1B**). This intersection of targets demonstrated the potential of Kirenol to exert its therapeutic effects specifically in the context of ovarian cancer.

### 4.2 Analysis of the PPI Network

To construct the PPI network, network analysis was performed on the 71 intersection targets using the STRING database. The PPI network's results were then imported into Cytoscape for additional in-depth examination. The resulting network, which showed the interactions between different proteins, had 328 edges and 66 nodes. By employing the cytoNCA module, specific key targets were identified based on several topological parameters, including Betweenness, Closeness, Eigenvector, LAC, Network, and Degree. As a result of this analysis, the top 8 key targets were determined to be mTOR, EGFR, IL6, JAK2, MAP2K1, MMP9, CDK4, and AR (Fig. **1C**). Furthermore, Table **1** presents the specific values of the 6 parameters corresponding to the 8 core targets. These key targets are of particular significance in the context of the potential action of Kirenol against ovarian cancer, as they are highly connected and influential nodes within the PPI network. Their identification provides valuable insights into the molecular mechanisms and pathways through which Kirenol may exert its therapeutic effects in ovarian cancer.

### 4.3 Enrichment Analysis

Functional enrichment analysis was performed on the 71 targets related to ovarian cancer, utilizing the DAVID biological information annotation database. This analysis included Gene Ontology (GO) and Kyoto Encyclopedia of Genes and Genomes (KEGG) analyses. Through GO analysis, it was discovered that Kirenol is associated with diverse molecular functions, including the activity of serine/threonine/tyrosine kinases, protein kinases, and protein binding. Kirenol is linked to cytosol, cytoplasm, and the plasma membrane in relation to cellular components. Furthermore, the examination revealed its participation in crucial biological activities, such as cellular growth, cellular movement, regulation of the cell cycle, processes of cell death, and the signaling pathway of tyrosine kinase (Fig.**1D**). Moreover, a barplot was generated to illustrate the top 10 enriched signaling pathways that may be affected by Kirenol in tumor-related contexts. Significantly, these pathways include pathways in cancer and the PI3K/AKT signaling pathway, among other pathways, offering valuable understanding into possible mechanisms by which Kirenol might exert its impacts in the context of tumor-related signaling (Fig.**1E**). In general, the functional enrichment analysis provided a thorough comprehension of Kirenol's participation in different molecular functions, cellular parts, and biological procedures, while also illuminating its potential influence on important tumor-associated signaling pathways. These findings contribute to the exploration of Kirenol as a promising candidate for ovarian cancer treatment.

### 4.4 Molecular Docking

To evaluate the binding interactions between the core genes and the active ingredients of Kirenol, molecular docking was utilized. The core genes were chosen by identifying the overlap between the top eight core genes (MTOR, EGFR, IL6, JAK2, MAP2K1, MMP9, CDK4, and AR) discovered in the protein-protein interaction (PPI) networks mentioned in Table **2**. Out of these core genes, Kirenol displayed the strongest affinity for CDK4, with a binding energy of -8.0 kcal/mol. The measurement of binding energy played a vital role in assessing the docking procedure. Binding energies equal to or less than -5 kcal/mol indicated the likelihood of binding interactions, while energies equal to or less than -7 kcal/mol indicated a favorable ability to bind. This analysis of molecular docking offers a valuable understanding of the potential interactions between the active components of Kirenol and the chosen core genes, with a particular emphasis on the significant binding affinity with CDK4. The results of this study enhance our comprehension of the molecular mechanisms that underlie the impacts of Kirenol on specific genes, providing potential implications for future therapeutic uses.

### 4.5 Kirenol Inhibits the Viability of Cell Lines

**Fig. (2):**
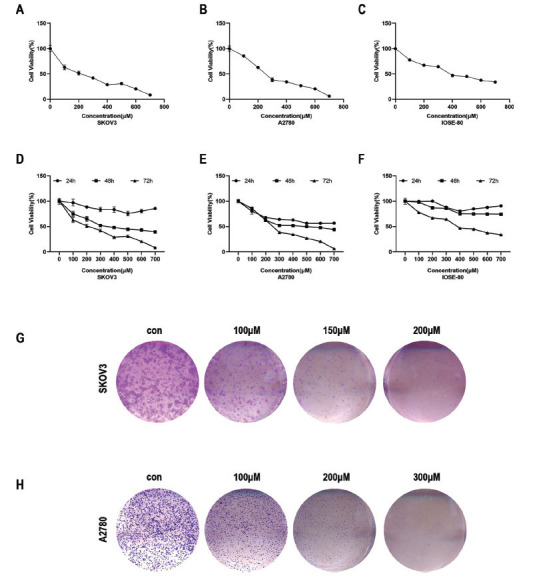
Kirenol has the ability to inhibit the proliferation of both regular ovarian epithelial cells and ovarian cancer cells. (**A-F**) Kirenol's IC50 value on SKOV3, A2780, and IOSE-80 normal ovarian epithelial cells. (**D-F**) By using the CCK8 assay, Kirenol prevents the growth of SKOV3, A2780, and IOSE-80 cells. (**G-H**) By preventing SKOV3 and A2780 cells from proliferating, Kirenol was found to inhibit colony formation.

After 72 hours of treatment with Kirenol, the IC50 values for SKOV3, A2780, and normal ovarian epithelial IOSE-80 cells decreased to 190 μmol/L, 259.1 μmol/L, and 395.4 μmol/L, respectively, resulting in decreased survival rates (Fig. **2A-C**). In the CCK-8 proliferation assay, Kirenol demonstrated an inhibitory effect on the growth of SKOV3, A2780, and normal ovarian epithelial IOSE-80 cells with increasing drug concentrations compared to the control group. However, normal ovarian epithelial IOSE-80 cells exhibited insensitivity to the inhibitory effect of Kirenol during the 24-hour and 48-hour periods. In contrast, SKOV3 and A2780 cells displayed dose- and time-dependent inhibition during this interval, with the inhibition rate reaching only around 60% at the maximum dose. Nevertheless, after 72 hours, the inhibition rate exceeded 90% at the maximum dose. While IOSE-80 cells also demonstrated dose-dependent inhibition after 72 hours, Kirenol exerted a stronger inhibitory effect on SKOV3 and A2780 cells. In the experiment on colony formation, when the concentrations of Kirenol were raised, there was a notable decrease in the number of colonies formed by SKOV3 and A2780 cells compared to the control group (Fig. **2D-H**). The results indicated that Kirenol has the ability to selectively and efficiently combat cancer, as it showed higher toxicity towards ovarian cancer cells (SKOV3 and A2780) while having minimal impact on normal ovarian epithelial cells (IOSE-80). The promising potential of Kirenol as a new therapeutic strategy for treating ovarian cancer is reinforced by the observed inhibitory effects on cell growth and colony formation, which vary depending on the dose and time.

### 4.6 Inhibition of Kirenol on Migration of Ovarian Cancer Cells

**Fig. (3):**
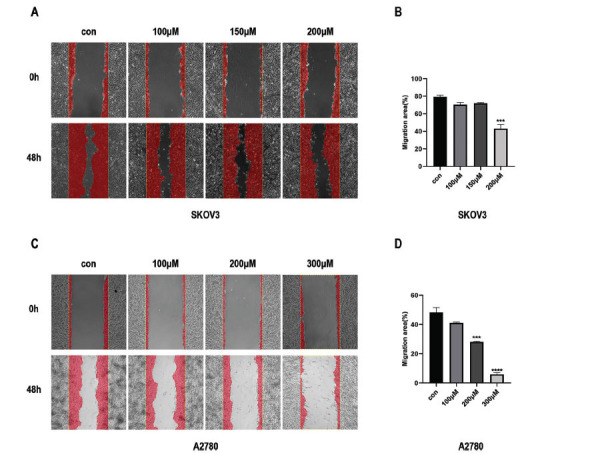
The ability of ovarian cancer cells to migrate can be inhibited by Kirenol. Based on the wound healing assay, Kirenol inhibits the movement of SKOV3 (**A-B**) and A2780 (**C-D**) cells. The experiment was independently conducted three times, with error bars representing the standard deviation. Compared to the control group, the statistical significance levels were as follows: *P < 0.05, **P < 0.01, ***P < 0.001, ****P < 0.0001.

Fig. (**3**) shows that the migration ability of SKOV3 and A2780 cells decreased in a dose-dependent manner when treated with Kirenol in the wound healing assay, compared to the control group (Fig. **3A-D**). This observation suggests that Kirenol effectively prevents these ovarian cancer cells from migrating. Furthermore, following the administration of Kirenol, the expression levels of MMP-2 and MMP-9 in SKOV3 and A2780 cells exhibited a notable reduction, as observed in a dose-dependent manner in the findings from the Western blot analysis. The decrease in the expression of these crucial matrix metalloproteinases implies that Kirenol might impede the cells' capacity to break down the extracellular matrix, consequently impacting their ability to migrate. The results indicated that Kirenol has the potential to inhibit metastasis, as shown by its ability to reduce the migratory capacity of ovarian cancer cells and decrease the expression of key factors involved in cell migration and invasion, including MMP-2 and MMP-9. This anti-metastatic effect adds to the compound's therapeutic value in targeting ovarian cancer and highlights its potential as a promising agent in the management of metastatic disease.

### 4.7 Kirenol Induces Cell Cycle Arrest and Apoptosis

**Fig. (4):**
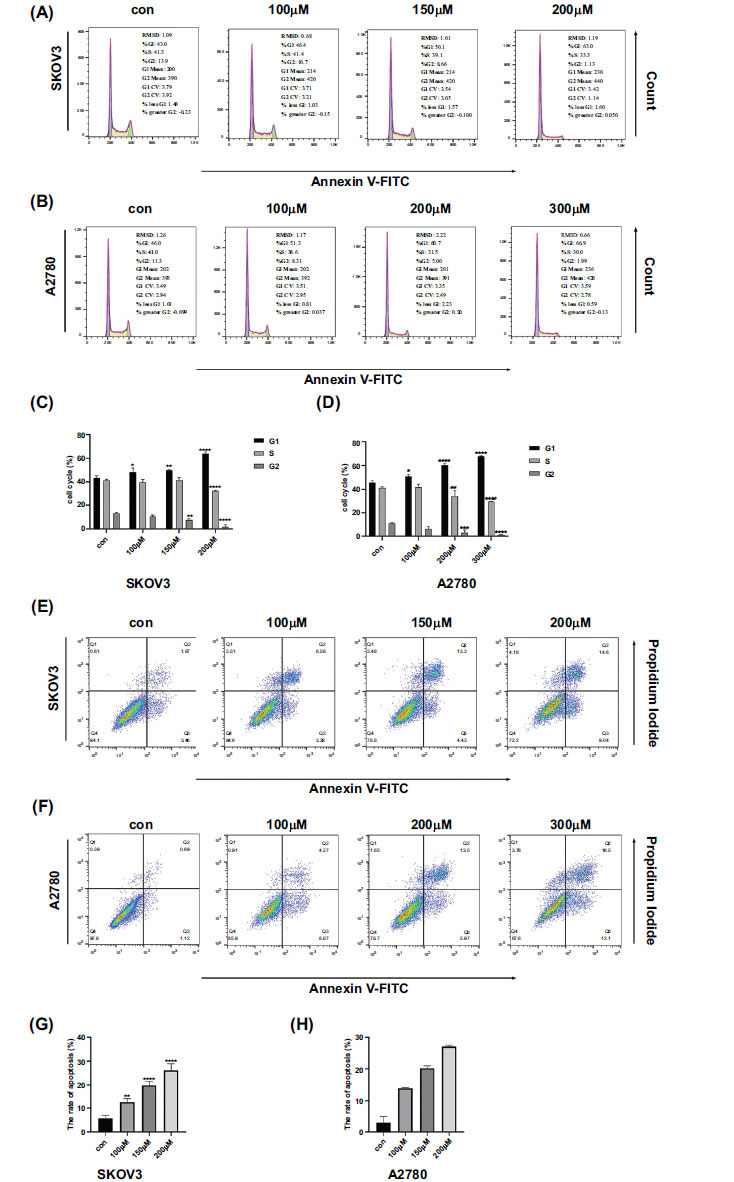
Kirenol induced apoptosis and halted the cell cycle. Kirenol inhibited the G0/1 phase of SKOV3 (**A, C**) and A2780 (**B, D**) cells. Compared to the control group, SKOV3 and A2780 cells exhibited an increase in the G1 phase and a decrease in the S phase. (A-D) The SKOV3 (**E, G**) and A2780 (**F, H**) cells undergo apoptosis in response to varying concentrations of Kirenol. The experiment was independently conducted three times, with error bars representing the standard deviation. Compared to the control group, the statistical significance levels were as follows: *P < 0.05, **P < 0.01, ***P < 0.001, ****P < 0.0001.

Kirenol causes cell cycle interruption and enhances apoptosis in SKOV3 and A2780 cells. The percentage of cells in the G0/G1 phase significantly increased when treated with Kirenol compared to the control group, whereas the percentage of cells in the S and G2/M phases decreased. This effect was more noticeable with higher concentrations of Kirenol, as shown in Fig. (**4A-D**). The results suggest that Kirenol disrupts the progression of the cell cycle, causing cells to accumulate in the G0/G1 phase and impeding their transition into the S and G2/M phases. Furthermore, Kirenol exhibited the capacity to trigger programmed cell death in both SKOV3 and A2780 cells, with the rate of apoptosis escalating in a manner that correlated with the concentration (Fig. **4E-H**). Confirmation of the apoptotic process was additionally supported through alterations in the protein expression of Bcl-2 and Bax. In both SKOV3 and A2780 cells, the use of Kirenol led to a significant decrease in Bcl-2 expression, while there was a notable increase in Bax expression. These changes were dose-dependent, as shown in Fig. (**5A-J**). These results indicated that Kirenol promotes apoptosis in ovarian cancer cells by modulating the expression of Bcl-2 and Bax, which are key regulators of the intrinsic apoptotic pathway. Additionally, its ability to induce cell cycle arrest further contributes to its anti-cancer effects. The results offer valuable perspectives on the possible ways in which Kirenol demonstrates its anti-cancer effects and endorse its potential as a promising therapeutic option for treating ovarian cancer.

### 4.8 Kirenol Functions through the PI3K/AKT/CDK4 Signaling Pathway

**Fig. (7):**
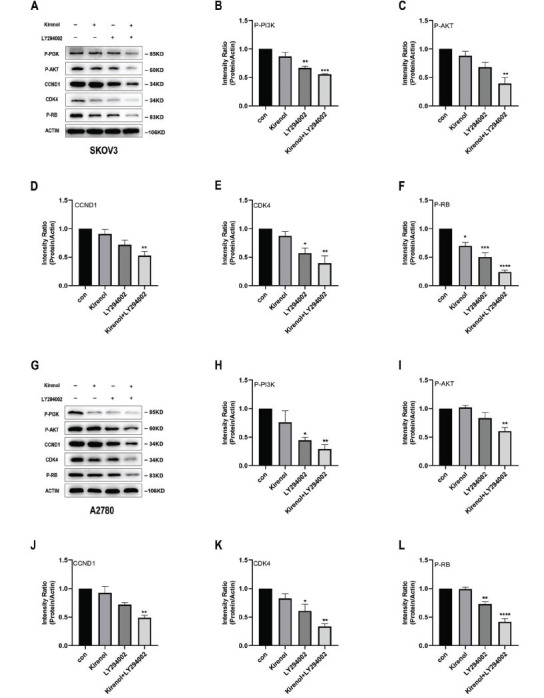
LY294002 functions through the PI3K/AKT/CDK4 signaling pathway. (**A-L**) LY294002 promoted the inhibition of Kirenol on PI3K/AKT and reduced the levels of P-PI3K, P-AKT, CCND1, CDK4, and P-RB. The experiment was repeated three times independently, and error bars indicate SD. *P < 0.05, **P < 0.01, ***P < 0.001, ****P < 0.0001 *vs*. the control group.

Western blot analysis revealed notable changes in the expression levels of various proteins in response to Kirenol treatment. In both SKOV3 and A2780 cells, the levels of P-PI3K, P-AKT, CCND1, CDK4, and P-RB exhibited a decrease, as shown in Fig. (**6A-P**). The discovery suggests that Kirenol may operate by impacting the PI3K/AKT/CDK4 signaling pathway, a widely recognized mechanism for regulating the cell cycle and stimulating cellular proliferation. In order to validate the involvement of the PI3K/AKT signaling pathway in Kirenol's mechanism, cells were subjected to treatment with LY294002, a specific inhibitor of the PI3K signaling pathway. LY294002 treatment resulted in the expected decrease of P-PI3K, P-AKT, CCND1, CDK4, and P-RB expression, indicating that the drug effectively inhibits the PI3K/AKT signaling pathway. These results are consistent with those observed under Kirenol treatment (Fig. **7A-L**). The possibility that Kirenol affects the function of ovarian cancer cells by changing the PI3K/AKT/CDK4 signaling pathway is indicated by all these findings. A decrease in important elements in this pathway, like P-PI3K, P-AKT, CCND1, CDK4, and P-RB, supports the idea that Kirenol could potentially inhibit this signaling cascade, providing new understanding of how it works and strengthening its potential as a hopeful treatment for ovarian cancer.

## 5 DISCUSSION

Ovarian cancer, a highly lethal gynecological malignancy worldwide [10, 11], has a survival rate below 45% after 5 years. Despite relying on surgery and chemotherapies [12], the significant challenges of metastases and chemotherapy resistance have impeded progress in reducing patient mortality and recurrence rates [13, 14]. Despite the advent of PARP inhibitors, resistance remains a significant concern for approximately 40-70% of patients [2]. Patients have been significantly burdened both physically and mentally due to the presence of drug resistance and harmful adverse reactions. Therefore, there is a pressing need to explore novel therapeutic agents, particularly those derived from Traditional Chinese Medicine (TCM), to enhance the treatment efficacy for ovarian cancer, ultimately improving patients' quality of life.

Kirenol, a diterpenoid characterized by the ent-pimarane structure [15], was initially employed in diverse anti-inflammatory treatments [16-18]. However, recent studies have demonstrated its tumor growth inhibitory properties against various human cancers, including gastric and thyroid cancers [8,9, 19]. Notably, its effects and molecular mechanisms against ovarian cancer remain unexplored in the current literature.

Our work is the first to examine the *in vitro* mechanisms underlying kirenol's inhibitory effect on ovarian cancer cells. After conducting a thorough analysis with multiple databases, including SwissTargetPrediction, Drugbank, String, Cytoscape, and DAVID biological information annotation, we identified CDK4 as a key target of Kirenol for the treatment of OC and predicted the associated pathways through which Kirenol exerts its inhibitory effects on OC. We also performed molecular docking and network pharmacological analysis. Notably, our Western blot results highlighted the significance of PI3K, AKT, and CDK4 as key genes associated with Kirenol treatment in OC. Phosphatidyl inositol-3-kinase (PI3K), one of the targets, is a member of a lipid kinase family that possesses both serine/threonine (Ser/Thr) kinase activity and phosphatidylinositol kinase activity. Akt, a protein kinase that phosphorylates serine and threonine residues, plays a vital role in governing various cellular processes, such as cell viability, movement, cell cycle advancement, and programmed cell death, under the influence of PI3K [20].CDK4 plays a pivotal role in regulating the G1-S transition and is inactivated by the cell cycle protein D1 (CCND1), forming a complex that phosphorylates the retinoblastoma (Rb) protein [21]. Additionally, CDK4 plays vital functions in facilitating the growth and spread of ovarian cancer cells [22]. Moreover, the suppression of CDK4 enhances the susceptibility of paclitaxel [23] in multidrug-resistant ovarian cancer cells. Crucially, earlier research has shown that the AKT pathway controls the expression of Cyclin D1/CDK4 [24, 25]. In our investigation, Kirenol decreased ovarian cancer cells' expression of CCND1 and phosphorylation of PI3K, AKT, and RB. Furthermore, the presence of LY294002, a PI3K/AKT pathway inhibitor, further enhanced these effects. Taking all these aspects into account, our investigation suggests that Kirenol has the potential to induce apoptosis in ovarian cancer cells by inhibiting the PI3K/AKT signaling pathway.

In this study, we utilized CCK-8 and colony-formation tests *in vitro* to assess the inhibitory effects of Kirenol on ovarian cancer cells, showcasing its potential to limit the proliferation of ovarian cancer cells. The CCK-8 test, employed to evaluate time and dose dependence, IC50 value variance, and cell survival rate, revealed the following key findings: the cytotoxic impact of Kirenol on normal ovarian epithelial cells was markedly lower compared to its effect on ovarian cancer cells. Kirenol demonstrated the ability to impede the proliferation of ovarian cancer cells at a dosage where the toxicity to normal ovarian epithelial cells was significantly lower than that observed in ovarian cancer cells. Additionally, the Wound healing assay demonstrated that Kirenol suppressed tumor cell migration. Significantly, Western blot tests demonstrated decreased levels of MMP2 and MMP9, pivotal elements in the spread of cancer, as they aid in breaking down the extracellular matrix and facilitating tumor metastasis [26]. Moreover, research on the cellular division process and programmed cell death indicated that Kirenol has the ability to induce a state of cell cycle arrest in ovarian cancer cells, specifically in the G0/G1 phase, and trigger apoptosis. Additional evidence for these effects was provided by the alterations in the levels of the pro-apoptotic protein Bax and the anti-apoptotic protein Bcl-2 subsequent to Kirenol treatment. Promoting apoptosis, Kirenol's potential as an anti-tumor agent is enhanced through the decrease in anti-apoptotic proteins and increase in pro-apoptotic proteins. In summary, our findings indicate that Kirenol influences the PI3K/AKT/CDK4 signaling pathway to exert its anti-cancer properties in ovarian tumors.

Despite these promising findings, our study has some limitations. We did not conduct animal experiments with Kirenol. Therefore, its clinical feasibility and availability require further investigations. To summarize, our research provides significant knowledge regarding the possible healing function of Kirenol in the treatment of ovarian cancer. The ability of the PI3K/AKT/CDK4 signaling pathway to hinder the growth, movement, and programmed cell death of ovarian cancer cells establishes a solid basis for further investigation and potential use in the management of ovarian cancer.

## CONCLUSION

Sum up, our research reports that Kirenol effectively suppresses the growth and movement of ovarian cancer cells by specifically targeting the PI3K/AKT/CDK4 signaling pathway. Moreover, it triggers cell cycle halt at the G0/G1 stage and enhances programmed cell death in ovarian cancer cells. The results emphasize the possible healing benefits of Kirenol as a hopeful contender in the creation of novel medications for the treatment of ovarian cancer. The identification of the effectiveness of Kirenol and its underlying molecular mechanisms offer valuable knowledge for future studies and open up new possibilities for investigating innovative therapeutic approaches in the fight against ovarian cancer.

## Figures and Tables

**Fig. (5):**
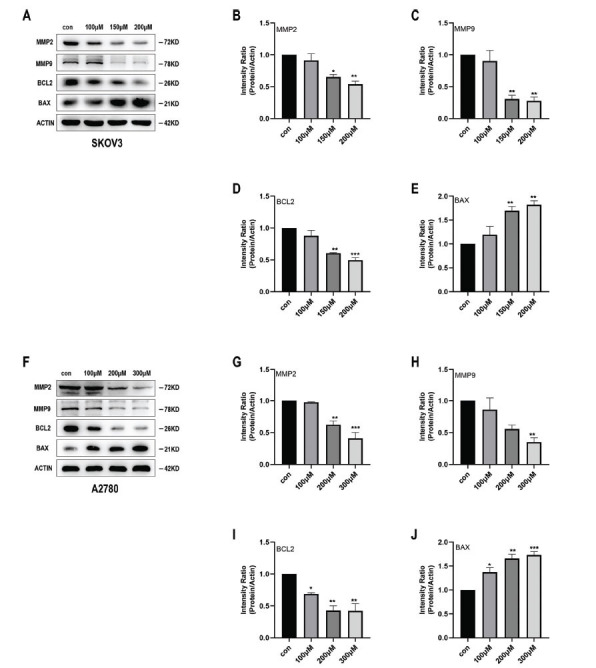
Kirenol decreased MMP2, MMP9, and Bcl-2 expression and increased Bax expression. (**A-J**) Protein levels of MMP2, MMP9, Bcl-2 and Bax, compared with those of the internal control ACTIN in SKOV3 (A-E) and A2780 (**F-J**) cells. Decreased MMP2, MMP9 and Bcl-2 expression and increased Bax expression were observed. The experiment was repeated three times independently, and error bars indicate SD. *P < 0.05, **P < 0.01, ***P < 0.001, ****P < 0.0001 *vs*. the control group.

**Fig. (6):**
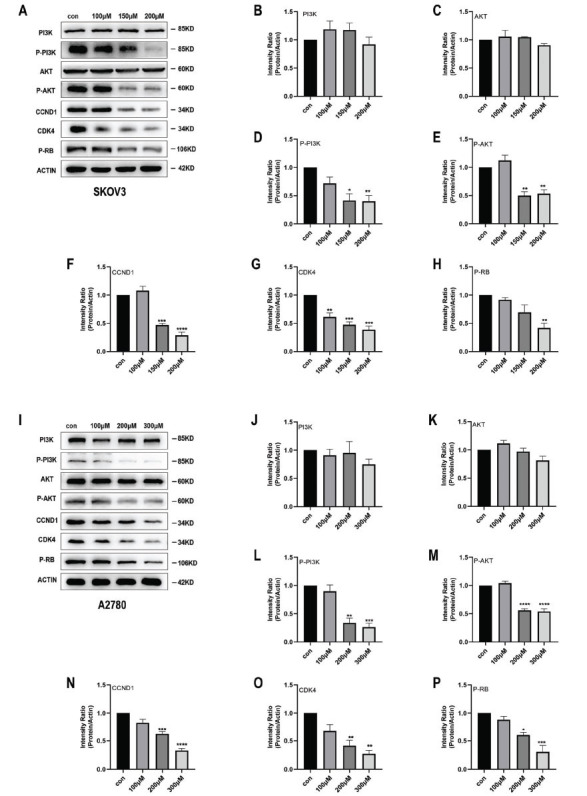
Kirenol functions through the PI3K/AKT/CDK4 signaling pathway. (**A-P**) Kirenol-treated cells did not decrease the total expression of PI3K and AKT compared to the control group, but the expression of P-PI3K, P-AKT, CCND1, CDK4, and P-RB was decreased. The experiment was repeated three times independently, and error bars indicate SD. *P < 0.05, **P < 0.01, ***P < 0.001, ****P < 0.0001 *vs*. the control group.

**Table 1:** The major parameters of the top 8 targets in the PPI network of Kirenol and OC.

**Name**	**Betweenness**	**Closeness**	**Degree**	**Eigenvector**	**LAC**	**Network**
MTOR	31.869913	0.913043	19	0.3124794	9.8947368	17.60307
EGFR	29.466666	0.875	18	0.2922655	9	15.48546
IL6	22.273088	0.84	17	0.289739	9.4117647	14.56142
JAK2	16.90563	0.777778	15	0.255906	8.266667	11.64624
MAP2K1	13.38023	0.75	14	0.240909	7.571429	10.02769
MMP9	8.929437	0.75	14	0.256305	8.857143	10.94662
CDK4	9.639683	0.724138	13	0.224392	7.692308	9.643939
AR	9.463564	0.724138	13	0.228772	7.692308	9.537374

**Table 2:** Molecular docking energy.

**Target**	**PDB ID**	**Compound**	**Affinity (kcal/mol)**
MTOR	1AUE	Kirenol	-6.3
EGFR	1IVO	Kirenol	-7.4
IL6	1ALU	Kirenol	-6.4
JAK2	2B7A	Kirenol	-7.1
MAP2K1	1S9J	Kirenol	-7.1
MMP9	1GKC	Kirenol	-6.6
CDK4	2W96	Kirenol	-8.0
AR	1E3G	Kirenol	-7.4

## Data Availability

The original contributions presented in the study are included in the article/Supplementary Material at: [https://www.scidb.cn/en/s/qquemi]. Further inquiries can be directed to the corresponding authors [H.Z.].

## LIST OF ABBREVIATIONS

OC: Ovarian Cancer
TCM: Traditional Chinese Medicine
PPI: Protein-Protein Interaction
FBS: Fetal Bovine Serum
DMSO: Dimethyl Sulfoxide
PBS: Phosphate-Buffered Saline
GI: Gastrointestinal Conditions
GO: Gene Ontology
KEGG: Kyoto Encyclopedia of Genes and Genomes
BC: Betweenness Centrality
CC: Closeness Centrality
DC: Degree Centrality
EC: Eigenvector Centrality
NC: Network Centrality
LAC: Local Edge Connectivity
OD: Optical Density
PMSF: Phenylmethylsulphonyl Fluoride
PAGE: Polyacrylamide Gel Electrophoresis
PVDF: Polyvinylidene Difluoride
SD: Standard Deviation
